# Susceptibility of microorganisms causing acute hand infections

**DOI:** 10.1371/journal.pone.0220555

**Published:** 2019-08-22

**Authors:** Nina Fuchsjäger, Herwig Winterleitner, Robert Krause, Gebhard Feierl, Horst Koch

**Affiliations:** 1 Division of Plastic, Aesthetic and Reconstructive surgery, Department of Surgery, Medical University of Graz, Graz, Austria; 2 Section of Infectious Diseases and Tropical Medicine, Department of Internal Medicine, Medical University of Graz, Graz, Austria; 3 Institute of Hygiene, Microbiology and Environmental Medicine, Medical University of Graz, Graz, Austria; Indiana University School of Medicine-Northwest, UNITED STATES

## Abstract

Hand infections are a common presentation at the emergency departments. Without knowing the source of infection clinicians are dependent on systematic reports on the bacterial spectrum and susceptibility tests of the specific infection in their patient community. This study was based on a retrospective chart review of patients presenting to our outpatient clinic with acute hand infections. We documented patient demographics, the etiology, location, culture tests of the infection and analyzed if certain bacteria could be cultured significantly more often in certain etiologies or in specific sites of the hand infection. Susceptibility tests were added. Bacterial swabs of 204 patients were analyzed. Overall *S*. *aureus* was found in 53% of all cases, in only one case revealed methicillin-resistant *S*. *aureus (*MRSA). There was no significant difference in the bacterial spectrum according to the etiology of the hand infections, except for animal bites where *Pasteurella multocida* was the dominating bacteria in 63% of all cases. Amoxicillin-clavulanic acid, fluoroquinolones, and piperacillin were effective against the main bacteria. Our study confirms the previously published antibiotic resistance reports and reinforces the current antibiotic treatment guidelines also in this western European population.

## Introduction

Hand infections form a major entity among clinical presentations at emergency departments, the plastic, surgical, or orthopedic outpatient clinics. In many cases, the primary cause of the infection may seem trivial. However, due to the unique anatomical features of the hand, the condition can easily spread which may result in severe functional impairment. Potential sequels include tissue necrosis, amputations, functional impairment and severe infections causing even death [1]. The clinical course and severity of acute hand infections depend on several factors: portal of entry of the pathogen, the site and depth of the infection, the etiology and the involved bacterial spectrum, the timing, and choice of treatment, as well as patient related factors such as age and comorbidities like diabetes mellitus or immunodeficiencies [2]. The optimal choice of the antibiotic treatment at an early stage, which is before the identification of the microorganism, is crucial for effective treatment. Clinicians, therefore, depend on reports of the most common spectrum of bacteria for acute hand infections and the updated reports of antimicrobial susceptibility of bacteria in their capture area.

The aim of our study was to investigate the bacterial etiology and the antimicrobial susceptibility pattern of microorganisms of community-acquired acute hand infections and to find possible associations with the site and etiology of the infection.

## Methods

A retrospective chart review was conducted. It comprised all in- and outpatients that were treated for infections of the hand and fingers at our institution over a 6-year period. The inclusion criteria were:

1) Infection of the hand and/or the fingers, and 2) Bacterial culture swabs collected from the site of the infection sent for bacterial cultures. The definition of hand infection was based on the assessment of the attending surgeon. Age and sex of the patients were documented as well as the final diagnosis and duration from the first medical contact to initiation of the initial therapy. Etiologies of hand and/or finger infections were categorized into 1) idiopathic, 2) trauma (any injury other than bite-injuries and minimal trauma), 3) animal bites, 4) minimal trauma (small and trivial skin lacerations), and 5) retained foreign bodies. According to the afflicted anatomical compartments, hand infections were categorized into different locations 1) Infection in a subcutaneous plane, 2) Paronychia, 3) Infection of flexor tendon sheaths or flexor tendons, and 4) Dorsal abscesses and infection after extravasation. The results of swab cultures and antimicrobial susceptibility tests were analyzed.

Wound swabs (Transystem, Copan Italia S.p.A., Brescia, Italy) were performed at the presentation and sent to the local microbiology lab (Institute of Hygiene, Microbiology and Environmental Medicine, Medical University of Graz, Austria) for bacterial culture and antimicrobial susceptibility testing.

We analyzed the results in a descriptive manner according to the etiology and localization of the wound infection and calculated p-values for the distribution of individual specimen for different etiologies and locations of the hand infections using Pearson’s chi-squared test. The retrospective single-center chart review was primarily coded to allow monitoring and then anonymised in a second step before the analysis, however, a formal ethical waiver would have been favorable but was not sought at that time.

## Results

### Culture results

A total of 296 patients were treated for acute infections of the hand and/or the fingers at our department, of which 204 patients had wound cultures taken at the presentation. The mean age of these patients was 46 years (range 18–94 years), 97 (48%) were female.

Of all 204 cultured specimens, 108 (53%) had growth of a single bacterium, while 93 (46%) had growth of more than one microorganism (Table 1). *S*. *aureus* was the most common microorganism and recovered in 109 specimens (53%). Methicillin-resistant *S*. *aureus (*MRSA*)* was found in only one specimen. Typical examples for mixed culture results included *S*. *aureus* with coagulase-negative staphylococci (3%), *S*. *aureus* with *Haemophilus parainfluenzae* (3%), *S*. *aureus* with *Enterobacter cloacae* (3%), *S*. *aureus with* viridans group streptococci (3%), *viridans group streptococci* with coagulase-negative Staphylococci (3%), and viridans group streptococci with *Prevotella* species (3%). Overall gram-positive microorganisms were cultured from 143 (70%) swabs, gram-negative strains from 21 (10%), and mixed gram-positive and gram-negative microorganisms from 40 (20%) of all swabs. Of all cultures, 73 (36%) showed a mixed aerobe and anaerobe infection, only one culture recovered a single anaerobe microorganism (0.5%).

**Table 1:** Characteristics of all specimens.

Characteristics	No. isolated (% of specimen)
**Total number of all specimens**	204
**Total number of cultured bacteria**	381
	
**No growth**	1 (0.5)
**One isolate**	108 (53)
**Two isolates**	41 (20)
**More than two isolates**	54 (27)
	
**Gram-positive**	142 (70)
**Gram-negative**	21 (10)
**Mixed Gram-positive and Gram-negative**	40 (20)
	
**Aerobes**	129 (64)
**Anaerobes**	1 (0.5)
**Mixed growth**	73 (36)
	
**All cultured bacteria**	381
***S*. *aureus***	109 (53)
***Coagulase-negative staphylococci***	30 (15)
***Viridans group streptococci***	25 (12)
***Prevotella species***	25 (12)
***Streptococcus agalactiae***	16 (8)
***Pasteurella multocida***	15 (7)
***Haemophilis parainfluenzae***	14 (7)
***Streptococcus pyogenes***	10 (5)
***Streptococcus intermedius***	11 (5)
***Enterobacter cloacae***	11 (5)
***Klebsiella spp*.**	8 (4)
***Escherichia coli***	6 (3)
***S*. *epidermidis***	5 (2)
***Enterococcus faecalis***	2 (1)
***Positive for any other bacteria or fungi***	94 (25)

### Bacterial spectrum related to etiology of infection

In the majority of all presentations (60%), patients were not aware of the cause of their hand infection. Of all known causes, injuries that were not caused by animal bites (22%) were the most common reason for the infection. Table 2 lists the distribution and bacterial spectrum of all presentations among the locations of the hand infection 1) idiopathic, 2) trauma, 3) animal bites, 4) minimal trauma (small and trivial skin lacerations), and 5) retained foreign bodies. Animal bites were significantly more often caused by *Pasteurella multocida* (12, 63%), followed by *Prevotella* species (3, 16%), *S*. *aureus* (2, 11%), *Haemophilus parainfluenzae* (2, 11%), and *Escherichia coli* (2, 11%).

**Table 2:** Distribution and bacterial spectrum according to different aetiologies of hand infections.

Total number of specimen n = 204	Idiopathic *n = 123*, 60%		Trauma *n = 45*, 22%		Animal bites *n = 19*, 9%		Small lacerations *n = 12*, 6%		Retained foreign bodies n = 5, 2%		P-value^a^
	*n*	%	n	%	n	%	n		n	%	
***S*. *aureus***	70	56	26	58	2	11	9	75	2	40	<0.01
***Coagulase-negative staphylococci***	21	17	5	11	2	11	1	8	1	20	>0.1
***Viridans group streptococci***	18	15	5	11	0	0	0	0	2	40	0.072
***Streptococcus agalactiae***	12	10	3	7	0	0	1	8	0	0	>0.1
***Prevotella species***	14	9	5	9	4	16	1	8	1	20	>0.1
***Streptococcus pyogenes***	8	7	2	4	0	0	0	0	0	0	>0.1
***Pasteurella multocida***	2	2	1	2	12	63	0	0	0	0	<0.01
***Streptococcus intermedius***	5	4	3	7	1	5	1	8	1	20	>0.1
***S*. *epidermidis***	3	2	1	2	0	0	1	8	0	0	>0.1
***Haemophilis parainfluenzae***	7	6	2	4	2	11	2	17	1	20	>0.1
***Escherichia coli***	4	3	1	2	1	5	0	0	0	0	>0.1
***Enterobacter cloacae***	9	7	1	2	1	5	0	0	0	0	>0.1
***Enterococcus faecalis***	2	2	0	0	0	0	0	0	0	0	>0.1
***Klebsiella spp*.**	7	6	1	2	0	0	0	0	0	0	>0.1

^a^ p-values were calculated using the Pearson’s chi-squared test.

### Bacterial spectrum related to localisation of infection

Table 3 lists the bacterial spectrum of all presentations of acute hand infections according to locations 1) Infection in a subcutaneous plane, 2) Paronychia, 3) Infection of flexor tendon sheaths or flexor tendons, and 4) Dorsal abscesses and infection after extravasation. Infections of the hand were most commonly in the subcutaneous plane (62%). The distribution of microorganisms in specific locations did not differ significantly from the overall distribution in hand infections, except for viridans group streptococci that was more often cultured from infections of the paronychia (9, 27%).

**Table 3:** Distribution and bacterial spectrum according to different localisations of hand infections.

Total number of all specimen n = 204	Infection in a subcutaneous plane n = 126, 62%		Paronychia n = 33, 16%		Infection of flexor tendon sheaths or flexor tendons n = 20, 10%		Dorsal abscesses and infection after extravasation n = 18, 9%		Not specified n = 7, 3%		P-value^a^
	*n*	%	*n*	%	*n*	%	*n*	%	*n*	%	
***S*. *aureus***	64	51	17	52	13	65	12	67	3	43	>0.1
***Coagulase-negative staphylococci***	14	11	8	24	4	20	2	11	2	29	>0.1
***Viridans group streptococci***	11	9	9	27	2	10	1	6	2	29	0.027
***Streptococcus agalactiae***	11	9	2	6	1	5	1	6	1	14	>0.1
***Prevotella species***	18	10	2	6	2	10	1	6	1	14	>0.1
***Streptococcus pyogenes***	8	6	1	3	1	5	0	0	0	0	>0.1
***Pasteurella multocida***	11	9	0	0	1	5	2	11	1	14	>0.1
***Streptococcus intermedius***	7	5	3	9	0	0	1	6	0	0	>0.1
***S*. *epidermidis***	3	2	0	0	1	5	0	0	1	14	>0.1
***Haemophilis parainfluenzae***	7	6	5	15	1	5	0	0	1	14	>0.1
***Escherichia coli***	2	2	1	3	2	10	1	6	0	0	>0.1
***Enterobacter cloacae***	8	6	2	6	1	5	0	0	0	0	>0.1
***Enterococcus faecalis***	1	1	0	0	1	5	0	0	0	0	>0.1
***Klebsiella spp*.**	6	5	1	3	0	0	0	0	1	14	>0.1

^a^ p-values were calculated using the Pearson’s chi-squared test.

### Antimicrobial susceptibility tests

Susceptibility tests for 253 bacteria cultured from 198 specimens were performed. We present susceptibility tests of bacteria with at least 10 identified isolates (Table 4). *S*. *aureus* isolates were 94–100% susceptible to most tested antimicrobials. Only 26% of all cultured *S*. *aureus* specimens were susceptible to penicillin and ampicillin. A methicillin-resistant strain was found in only one case. *Streptococcus agalactiae* was 100% susceptible to most tested antimicrobials, 94% were susceptible to erythromycin, 85% to trimethoprim-sulfamethoxazole, 35% to tetracycline. *Pasteurella multocida* was susceptible to all tested antimicrobials. *Streptococcus pyogenes* was susceptible to most tested antimicrobials, in 89% of all cases to clindamycin and erythromycin, in 67% of all cases to other tested macrolides, and in 60% of all cases it was susceptible to trimethoprim-sulfamethoxazole. Coagulase-negative staphylococci were 100% susceptible to all aminoglycosides, ciprofloxacin, tetracycline, vancomycin, gentamicin, and fusidic acid, and 75% were susceptible to oxacillin. *Enterobacter cloacae* was 100% susceptible to third-generation cephalosporins and all other tested antimicrobials, but not susceptible to second-generation cephalosporins and amoxicillin-clavulanic acid. As for the less common bacteria, viridans group streptococci was 100% susceptible to all tested penicillins and cephalosporins, ciprofloxacin, trimethoprim-sulfamethoxazole, vancomycin, teicoplanin and linezolid, and 85–87% susceptible to tetracycline, erythromycin, and clindamycin (not shown in table). No susceptibility tests were available for *Prevotella* species.

**Table 4:** Susceptibility of most commonly found bacteria.

	Bacteria, % susceptible				
	*S*. *aureus*	*S*. *agalactiae*	*P*. *multocida*	*Enterobacter cloacae*	*H*. *parainfluenzae*
**Total number of isolates**	**112**	**17**	**15**	**10**	**10**
**Pen**	23	100	100	-	-
**Oxa**	100	-	-	-	-
**Amp**	23	100	100	0	100
**Amo/clav**	100	100	100	10	100
**Mezl**	-	-	100	100	-
**Pip**	100	100	100	100	-
**Cefac**	100	100	-	0	100
**Cefu**	100	100	100	0	90
**Cefal**	100	100	100	0	100
**Cefoxit**	-	-	100	11	-
**Cefotax**	100	100	100	100	100
**Ceftazidi**	-	-	100	100	-
**Cefepim**	-	-	100	100	-
**Ceftr**	100	100	100	-	-
**Mero**	-	-	100	100	-
**Imi**	100	100	100	100	-
**Gm**	97	-	100	100	100
**Tobra**	97	-	-	100	-
**Ami**	94	35	-	100	90
**Cipr**	99	100	100	100	-
**Mox**	100	-	-	-	-
**Oflo**	100	100	-	100	100
**Levo**	100	-	-	-	-
**Ery**	96	94	-	-	100
**Azit**	-	100	-	-	-
**Josa**	-	100	-	-	-
**Clari**	-	100	-	-	-
**Tet**	94	35	-	100	90
**Mino**	100	-	-	-	-
**Clind**	98	100	-	-	-
**Vanc**	100	100	-	-	-
**Sxt**	99	85	100	100	70
**Fos**	100	100	-	-	-
**Teico**	100	100	-	-	-
**Fusi**	99	-	-	-	-
**Rif**	100	-	-	-	100
**Lin**	100	100	-	-	-

Pen, penicillin; Oxa, oxacillin; Amp, ampicillin; Amo, Amoxicillin-clavulanic acid; Mezl, mezlocillin; Pip, piperacilin; Cefac, cefaclor; Cefu, cefuroxime; Cefal, cefalexin; Cefoxit, cefoxitin; Cefotax, cefotaxime; Ceftazidi, ceftazidime; Cefepim, cefepime; Ceftr, ceftriaxone; Mero, meropenem; Imi, Imipenem; Gm, gentamicin; Tobra, tobramycine, Ami, amikacin; Cipr, ciprofloxacin; Mox; moxifloxacin; Oflo, ofloxacin; Levo, levofloxacin; Ery, erythromycin, Azit, azithromycin, Josa, josamycin; Clari, clarithromycin.

Tet, tetracycline; Mino, minocycline; Clind, clindamycin; Vanc, vancomycin; Sxt, trimethoprim-sulfamethoxazole; Fos, fosfomycine; Teico, teicoplanin; Fusi, fusidic acid; Rif, rifampicin, Lin, linezolid; -, not tested

## Discussion

After the analysis of 204 specimens we conclude the following: 1) in our capture area, *S*. *aureus* is the dominating bacterium in most of acute non-bite hand infections and there is very low incidence of MRSA. 2) The dominating bacterium found in cultures from animal bites is *Pasteurella multocida*. 3) The site of the hand infection does not seem to influence the bacterial spectrum. 4) Amoxicillin-clavulanic acid, cephalosporins, and fluoroquinolones are highly susceptible and may be considered for recommendations of an empiric first-line antibiotic treatment. Other antibiotics show some variations in susceptibility patterns across various pathogens of the infection. 5) While *Pasteurella multocida* and viridans group streptococci seem to be highly susceptible to the indicated antibiotics, *Streptococcus agalactiae*, S. *pyogenes*, and coagulase-negative staphylococci are resistant to a number of antibiotics.

Our results are important as they add to the current reports on bacterial spectrum and susceptibility of microorganisms in acute hand infections. These reports serve as an important basis for the decision making of the initial antibiotic treatment of acute hand infections. A tailored surgical intervention and antimicrobial treatment are the mainstay for patients presenting with acute hand infection, especially in severe and advanced cases. Besides reporting the bacterial spectrum, we attempted to find possible predictors to narrow the suspected bacterial spectrum in our patients.

There are few recent reports on the epidemiology of patients presenting with acute hand infections [1, 3]. Anwar and colleagues published the results of a retrospective chart review with data from 76 patients with hand infections admitted to the plastic surgery unit at a general hospital in the United Kingdom. Very similar to our results the most common of all bacteria was *S*. *aureus*, followed by *Streptococcus*, *Pasteurella multocida* in bite-wounds, and no case of a methicillin-resistant *S*. *aureus*. Of note, of the infections with *S*. *aureus*, at least 90% were community acquired. Another study published by Fowler and colleagues in the United States showed a very different picture. Here, MRSA was found in over 50% of all outpatients with hand infections [4]. The reason for this may be that in contrast to the US the incidence of community acquired-MRSA infections in Europe is much lower; however, numbers have been raising in the past decade [5,6, 7, 8, 9]. It is important to note the differences in the spectrum and etiology of acute skin and soft tissue infections. While patients with diabetic food infections show a much higher number of polymicrobial growth and prevalence of MRSA [7] the spectrum susceptibility of acute hand infections in mostly healthy adults seems to reflect the common microorganisms in the community.

One limitation of our study may be that we do not have complete information regarding comorbidities. Next to the etiology, these could have turned out to influence the bacterial spectrum in hand infections and be an indicator for the culprit bacteria in our patients, as shown in one other study [4]. Also, the results represent the bacterial spectrum of wound swabs of a common surgical outpatient clinic. They were taken under non-sterile conditions, and it is possible that common contaminants like *S*. *aureus*, coagulase-negative staphylococci *and Pseudomonas* genera may have originated from the bacterial skin flora [8, 10].

Our study does not differ relevantly from the current antibiotic resistance reports and hence reinforces the commonly used antibiotic treatment guidelines [11]. The low incidence of MRSA in comparison to other skin and soft tissue infections is an interesting aspect that was already shown in European studies a few years ago, and does not seem to have changed.

## Supporting information

S1 Dataset Acute hand infections for publication. (XLSX)

S2 Dataset Acute hand infections for publication. (XLSX)
